# Sulfate Reduction for Bioremediation of AMD Facilitated by an Indigenous Acid- and Metal-Tolerant Sulfate-Reducer

**DOI:** 10.4014/jmb.2001.01012

**Published:** 2020-03-09

**Authors:** Hai Thi Nguyen, Huong Lan Nguyen, Minh Hong Nguyen, Thao Kim Nu Nguyen, Hang Thuy Dinh

**Affiliations:** 1 VNU Institute of Microbiology and Biotechnology (IMBT), Vietnam National University Hanoi, 144 Xuan Thuy, Cau Giay, Hanoi, Vietnam; 2 Hanoi University of Science and Technology (HUST), 1 Dai Co Viet Road, Hanoi, Vietnam

**Keywords:** Acid mine drainage (AMD), acid-tolerant sulfate-reducing bacteria, *Delta-proteobacteria*, *Desulfovibrio* sp., heavy metal removal

## Abstract

Acid mine drainage (AMD) has been a serious environmental issue that threatens soil and aquatic ecosystems. In this study, an acid-tolerant sulfate-reducing bacterium, strain S4, was isolated from the mud of an AMD storage pond in Vietnam via enrichment in anoxic mineral medium at pH 5. Comparative analyses of sequences of the 16S rRNA gene and *dsrB* gene involved in sulfate reduction revealed that the isolate belonged to the genus *Desulfovibrio*, and is most closely related to *Desulfovibrio oxamicus* (with 99*%* homology in 16S rDNA sequence and 98% homology in *dsrB* gene sequence). Denaturing gradient gel electrophoresis (DGGE) analyses of *dsrB* gene showed that strain S4 represented one of the two most abundant groups developed in the enrichment culture. Notably, strain S4 was capable of reducing sulfate in low pH environments (from 2 and above), and resistance to extremely high concentration of heavy metals (Fe 3,000 mg/l, Zn 100 mg/l, Cu 100 mg/ l). In a batch incubation experiment in synthetic AMD with pH 3.5, strain S4 showed strong effects in facilitating growth of a neutrophilic, metal sensitive *Desulfovibrio* sp. strain SR4H, which was not capable of growing alone in such an environment. Thus, it is postulated that under extreme conditions such as an AMD environment, acid- and metal-tolerant sulfate-reducing bacteria (SRB)- like strain S4 would facilitate the growth of other widely distributed SRB by starting to reduce sulfate at low pH, thus increasing pH and lowering the metal concentration in the environment. Owing to such unique physiological characteristics, strain S4 shows great potential for application in sustainable remediation of AMD.

## Introduction

Mining activities bring sulfidic ores into contact with oxygen, resulting in oxidation of pyrite-containing rocks to form sulfuric acid and dissolve ferrous iron as well as other metals in the ores, leading to the production of acidic wastewater with high metal content, called acid mine drainage (AMD). Thus, AMD is characterized by extremely low pH (2–3), containing various heavy metals at high concentration (up to 100 ppm) and is very toxic to aquatic life or soil ecosystems close to mining areas [1].

Sulfate-reducing bacteria (SRB) are known to be capable of reducing sulfate to sulfide, therefore they are used for metal removal in sulfate-reducing bioreactors, the passive treatment technology for AMD [2]. Metal ions such as iron, lead, copper, nickel, cadmium and zinc can be easily precipitated in the form of metal sulfides and thereby are removed from the wastewater. Some other metals and metalloids, *e.g*., molybdenum, arsenic and antimony, can form more complex insoluble sulfide minerals [3].

In the sulfate-reducing bioreactor for AMD treatment, acid-tolerant SRB would have an advantage at the initial phase since they can promote the sulfate reduction at low pH, raising the pH to more favorable values for neutrophilic SRB [4]. However, with the native physiological trait of producing sulfide during the sulfate metabolism, SRB tend to grow under neutral or alkali conditions, rather than in acidic condition [5]. Indeed, it has been found that sulfate reduction rate significantly decreased when pH dropped from 6 to 4 [6]. Nevertheless, the growth of SRB under acidic condition was observed for some strains of SRB, such as *Desulfosporosinus* spp., a spore-forming group within the family *Peptococcaceae* in the phylum *Firmicutes* [7-9]. A rare case was the acid- tolerant *Desulfovibiro* sp. strain TomC, a representative of SRB within the class *Delta-proteobacteria*, isolated from the acidic waste of a gold-mining site in Siberia [10]. It was noteworthy that this strain was shown to be capable of growing under extreme condition of AMD, *e.g*., pH of 2.5 and high concentrations of heavy metals [10].

In this study, another acid-tolerant SRB of the genus *Desulfovibrio*, strain S4, was isolated from the mud of an AMD storage pond in Vietnam. This strain was able to reduce sulfate at pH as low as 2 and tolerate high concentrations of various heavy metals, including Zn^2+^ and Cu^2+^, which are highly toxic to microbial cells. Under the extreme environmental conditions in an AMD environment, strain S4, owing to these specific characteristics, would facilitate the growth of other SRB that are more sensitive to low pH and high metal contents, and would therefore be of interest for application in AMD bioremediation.

## Materials and Methods

### Enrichment and Isolation of Acid-Tolerant SRB

The enrichment of SRB was carried out in selective anoxic lactate-sulfate (LS) medium with the following composition: Na_2_SO_4_, 4 g; NaCl, 1 g; MgCl_2_•6H_2_O, 0.4 g; CaCl_2_•2H_2_O, 0.15 g; KCl, 0.5 g; MgSO_4_•7H_2_O, 0.25 g; NH_4_Cl, 0.25 g; KH_2_PO_4_, 0.2 g; distilled H_2_O, 1 L [11]. The medium was sterilized for 20 min at 121°C, and afterward flushed with a gas mixture, N_2_:CO_2_ 90:10 (vol/vol) for 5 min to remove oxygen. After cooling down to room temperature, the following supplements were added (into one liter of medium): 30 ml of 1 M NaHCO_3_; 1 ml of thiamine solution (0.1 g/l in phosphate buffer 50 mM, pH 3.7, filter sterilized); 1 ml of cyanocobalamin solution (0.05 g/l deionized water, filter sterilized); 1 ml of trace element mixture [11]; 1 ml of 1 M Na_2_S; 10 ml of 1 M sodium lactate. The pH was adjusted to 5.0 by using sterile 1 M HCl. Afterward, the medium was dispensed into 100-ml sterile serum bottles, tightly sealed with rubber stoppers under N_2_:CO_2_ 90:10 (vol/vol) gas stream. Iron nails were put in the bottles to help remove the dissolved sulfide which is critical for the bacteria to grow at low pH. The initial source of SRB was acidic mud from a wastewater receiving pond at a tin processing factory in Tuyen Quang, Vietnam (21°48'48.8"N 105°12'40.0"E). The enrichment cultures were incubated at 28°C in the dark and transferred into fresh LS medium every 7 days. In total, three successive transfers were carried out before the isolation of pure cultures was performed.

The isolation of acid-tolerant SRB was carried out via serial dilution in anoxic semi-liquid agar (1%, w/v) LS medium with pH 7. The agar tubes were incubated at 28°C for 1–2 weeks until blackish-to-gray colonies of SRB were visible. Single colonies were picked by glass capillaries and transferred into fresh liquid anoxic LS medium.

### Physiological Characterization

The bacterial sulfate reduction tests were performed in gas tight, sealed serum bottles or screw cap glass tubes containing anoxic liquid media. The bacterial cultures were incubated at 28°C in the dark under static condition and the bacterial sulfate reduction was quantified within 15 days of incubation.

Experiment with the pH-dependent sulfate reduction was carried out in liquid LS medium with pH adjusted from 4 to 7 by using 1 M HCl.

The sulfate reduction performance of strains at different pH from 2 to 7 with alternative electron donors was carried out in sulfate (28 mM)-containing medium supplemented with lactate or ethanol (20 mM).

To examine their resistance to heavy metal ions, the bacteria were cultivated in anoxic LS medium, supplemented with one of the following metal ions: Fe^2+^ (at the concentrations of 60, 200, 400, 500, 800, 2,000, and 3,000 mg/l), Zn^2+^ (at the concentrations of 20, 50, 70, 100 mg/l), and Cu^2+^ (at the concentrations of 10, 20, 30, 40, 50, 100 mg/l). To avoid metal ion precipitation, sodium ascorbate (1 mM) was used as a reducing agent instead of sulfide (1 mM). The bacterial sulfate reduction in the presence of different metal ions was evaluated.

### Batch Incubation with Neutrophilic SRB Species

To demonstrate the role of acid-tolerant strain S4 in supporting the growth of other neutrophilic SRB species that are not able to grow in low pH environment, a batch incubation experiment was carried out with co- inoculation of this strain and a common neutrophilic *Desulfovibrio* sp. strain SR4H from Vietnam Type Culture Collection (VTCC 11270). As controls, these two strains were cultured separately. The synthetic AMD [12] with pH 3.5 was used as culturing medium, containing 20 mM lactate and different metal ions, namely Fe^2+^ (380 mg/l), Zn^2+^ (20 mg/l) or Cu^2+^ (8.3 mg/l). Active culture of strain SR4H was added into the culture of strain S4 pre-grown for 0, 3, 6, 9, 12, and 15 days. Sulfate reduction in the incubating bottles was measured in single- or mixed cultures.

### Analyses of 16S rDNA

The genomic DNA of bacterial isolates was extracted following Marmur *et al*. [13] with slight modifications. Briefly, cells from 5-day-old cultures were collected by centrifugation at 9,000 ×*g* for 10 min, and then were re- suspended in 0.5 ml of 5 mM EDTA (pH 8). Afterward, the cells were treated with lysozyme (50 μl of 40 mg/ml) at 37°C for 3 h, then with SDS (50 μl of 20%, w/v) and proteinase K (50 μl of 4 mg/ml) at 55°C for 1 h. Extraction was performed by adding an equal volume of phenol-chloroform-isoamyl alcohol (25:24:1, v/v), mixing and centrifugation at 15,600 ×*g* for 15 min at room temperature. Chromosomal DNA was precipitated using 2 volumes of cold 2-propanol, incubated at room temperature for one hour, then collected by centrifugation at 15,600 ×*g*. Finally, DNA was rinsed with 70% ethanol, air-dried and dissolved in 50 μl of sterile distilled water.

The 16S rRNA gene was amplified using primers 27F (5’-AGAGTTTGATCCTGGCTCAG-3’) and 1492R (5’- GGTTACCTTGTTACGACTT-3’) [14]. The reaction mixture (50 μl) contained 5 μl of 10× reaction buffer (0.2 M Tris-HCl pH 8.3, 0.25 M KCl, 20 mM MgCl2), 20 nmol of each deoxyribonucleotide, 50 pmol of primer, 2.5 U of *Taq* DNA polymerase (Promega), and 1 μl of template DNA (10 ng ml^-1^). Thermocycles for the PCR included 5 min at 95°C, followed by 30 cycles of 95°C for 30 sec, 52°C for 30 sec, 72°C for 1 min, and a final extension at 72°C for 7 min. The PCR products were then analyzed by electrophoresis on 1% (w/v) agarose gel. Prior to sequencing, the PCR products were purified with a PCR purification kit (Qiagen), and the sequencing was performed on an ABI 3110 Avant Applied Biosystems sequencer (ABI, USA).

The 16S rDNA sequences were compared with related sequences available on the GenBank database by using the BLAST Search tool. The alignment of sequences was performed with the 16S rDNA sequences by using the CLUSTAL_X program, version 1.8, and a phylogenetic tree was reconstructed using the neighbor-joining method [15]. Topography of the reconstructed tree was evaluated by bootstrap analysis with 1000 replicates [16].

### DGGE Analyses of the dsrB Gene Fragments

*dsrB* gene fragments (350 bp) were amplified using the primer pair DSRp2060F-GC (5’-CAACATCGTYCA YACCCAGGG-3’) [17] and DSR4R (5’-GTGTAGCAGTTACCGCA-3’) [18]. A GC-clamp of 40 nucleotides [19] was added to the 5’-end of the primer DSRp2060F to stabilize the migration of PCR products in denaturing gels during electrophoresis. The amplification was achieved with a “touchdown” thermocycle [17], comprising of a denaturation step at 95°C for 5 min, followed by 20 cycles of denaturation for 40 sec at 95°C, annealing for 40 sec at “touchdown” temperature gradually decreased from 65°C to 55°C in 20 cycles, and elongation for 1 min at 72°C. Afterward, the PCR was followed by 15 cycles of 95°C for 40 sec, 55°C for 40 sec, and 72°C for 1 min. The ampliﬁcation was completed by a ﬁnal elongation step at 72°C for 10 min and ended at 4°C.

DGGE was performed on 8% polyacrylamide gel containing a linear gradient of 30% to 70% denaturants (urea and formamide). The electrophoresis was carried out in the Bio-Rad DCode system at 150 V and 60°C for 6 h. After the electrophoresis, the gel was stained in ethidium bromide solution (5 mg/ml) for 30 min and washed in deionized water for 15 min before taking images on the Geldoc system (Bio-Rad) under UV light. Prominent bands were excised and emerged in 100 μl deionized water overnight at 4°C for the DNA elution. Amplification reaction was performed with the eluted DNA as template and the primer pair DSRp2060F and DSR4R. The thermocycling comprised of 35 cycles of 95°C for 40 sec, 55°C for 40 sec, 72°C for 1 min. The PCR products were then checked by electrophoresis on 1% (w/v) agarose gel, purified and sequenced. The *dsrB* gene sequences were compared with sequences available on the GenBank database by using the BLAST Search tool.

### Chemical Analyses

Total iron content was measured by using standard method for the examination of water, wastewater and sludge [20]. The chemical principle of method lies on the reaction of ferrous iron with O-phenanthroline to produce a purple complex (at pH in the range 3–9), which can be quantified at the wavelength of 510 nm.

The concentrations of metal ions Cu^2+^ and Zn^2+^ were determined by flame atomic absorption spectrometry [21]. Prior to the measurement, samples were filtered to remove biomass and metal precipitates, then treated with 1M HNO_3_ to drop the pH to 1.

Sulfate was determined micro-gravimetrically as BaSO4 precipitated from 1 ml cell free samples reacted with the same volume of 0.2 M BaCl2. The formed BaSO4 was filtered by using a 0.2 μm membrane filter, then dried to constant weight and weighed.

All experiments were carried out in triplicates. Experimental data were processed using Microsoft Excel software (average and standard deviation functions, the standard deviation calculated using the “n-1” method) and graphed by using Sigmaplot 14 software.

### Nucleotide Sequence Accession Numbers

The 16S rDNA sequences of SRB isolates obtained in the study, strains S4 and S10, were deposited at DDBJ database with the accession numbers LC186051 and LC469350, respectively. The *dsrB* gene sequences of these strains were deposited at GenBank with the accession numbers of MN792774 for strain S4 and MN792773 for strain S10.

## Results

### Enrichment and Isolation of Acid-Tolerant Sulfate-Reducing Bacteria

**Fig. 1:**
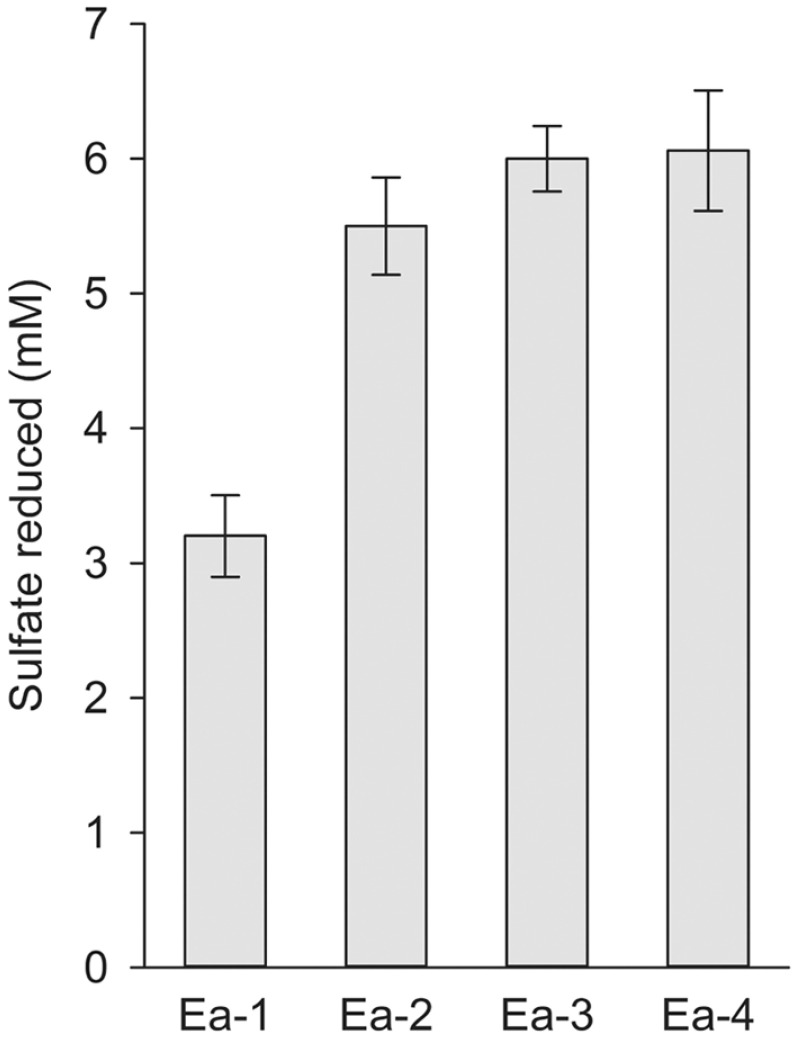
Sulfate reduction in the enrichment cultures of SRB at low pH (pH 5) after 7 days of cultivation. Ea-1 was the started enrichment incubation; Ea-2 through Ea-4 were the enrichment cultures at the 1^st^ through the 3^rd^ transfers.

The enrichment of SRB was established in LS liquid medium at pH 5 using a mud sample collected at an AMD storage pond as the inoculum. The obtained results (Fig. 1) showed that sulfate reduction in the enrichment culture after the first sub-culturing step (Ea-2) was significantly higher than that in the first incubation (Ea-1). The sulfate reduction became relatively stable, reaching ~ 6 mM at day 7 in the next two sub-culturing steps (Ea-3 and Ea-4). Accordingly, the cultures turned totally black due to significant metal sulfide precipitation (not shown).

**Fig. 2:**
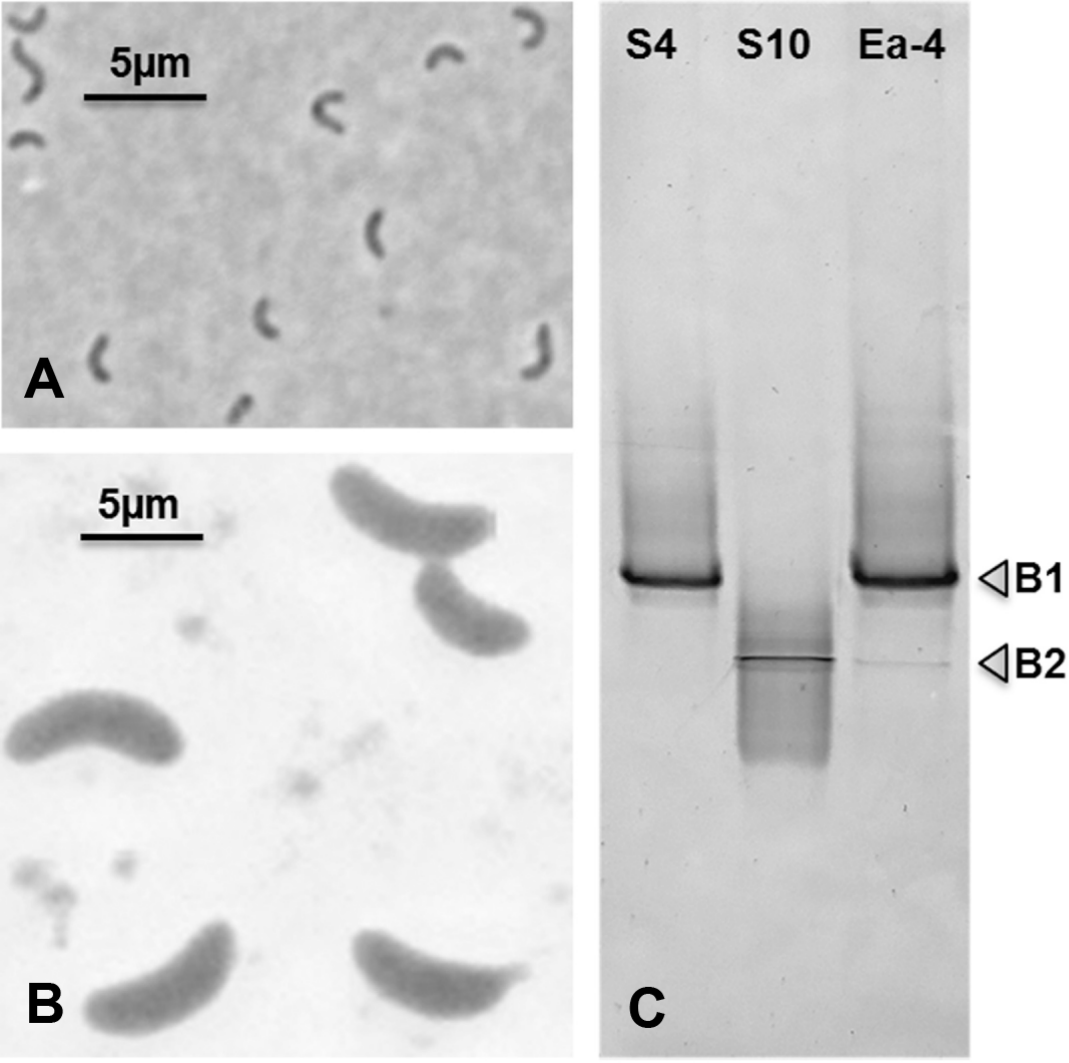
Cell morphology of the SRB isolates, strain S4 (A) and strain S10 (B) observed under phase contrast microscopy. DGGE analysis of *dsrB* gene fragments retrieved from these isolates in comparison with the enrichment culture Ea-4 (**C**). The DGGE was performed on 8% polyacrylamide gel containing linear gradient of 30% to 70% denaturants (urea and formamide). It was shown that these isolates represented the two most abundant SRB groups in the enrichment culture Ea-4.

Isolation of SRB was performed with the enrichment culture Ea-4 after 7 days of incubation. Two SRB strains, designated as S4 and S10, were obtained. Cells of strain S4 were small, curved, 0.6–0.7 × 2–3 μm in size, and actively moving (Fig. 2A), whereas strain S10 comprised of big vibrio, slowly moving cells of 2–2.3 × 5–8 μm in size (Fig. 2B).

DGGE analyses of *dsrB* gene fragments (350 bp) derived from the enrichment culture Ea-4 showed that it contained two main bands, B1 and B2 (Fig. 2C). The nucleic acid sequences of these bands were affiliated to the *dsrB* gene of the newly isolated strains S4 and S10. Evidentially, the low pH condition was strongly selective for SRB, thus leading to two dominant groups of SRB in the enrichment culture Ea-4.

**Fig. 3:**
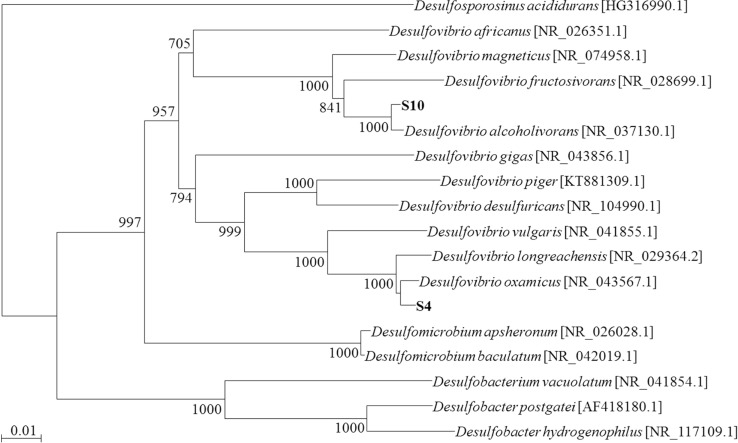
Phylogenetic tree reconstructed by 16S rDNA sequence analyses showing taxonomic positions of the two isolates S4 and S10 among other SRB. The tree was reconstructed using neighbor-joining method and its topography was evaluated by bootstrap analysis with 1000 replicates.

Comparative analysis of nearly full-length 16S rDNA sequences of the isolates S4 and S10 revealed that both strains belong to the genus *Desulfovibrio.* In detail, strain S4 was most closely related to *D. oxamicus* (99% sequence homology) and strain S10 to *D. alcoholivorans* (99% sequence homology) (Fig. 3). In agreement with the phylogenetic affiliation based on 16S rDNA sequences, *dsrB* gene sequences of strains S4 and S10 were 98% homologous with that of *D. oxamicus* and *Desulfovibrio* sp., respectively.

### Acid Tolerance of the Isolates

**Fig. 4:**
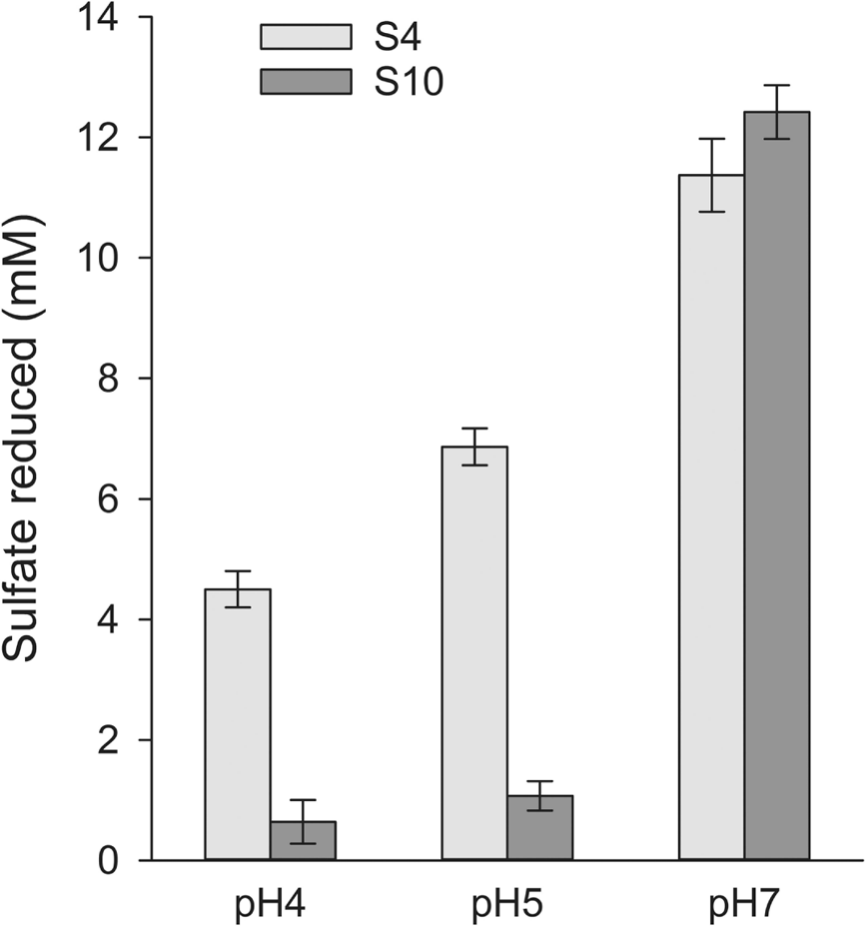
pH-dependent sulfate reduction by strains S4 and S10 in lactate-sulfate medium.

Acid tolerance was the most important characteristic that needed to be investigated of the new SRB isolates. Thus, strains S4 and S10 were cultivated in LS medium with pH adjusted at three different values of 4, 5 or 7. It can be seen from Fig. 4 that both strains reduced sulfate most effectively at pH 7. At acidic pH values of 5 and 4, strain S10 significantly decreased sulfate reduction activity, whereas strain S4 remained to reduce sulfate with a rate equal to 40−60% of that at pH 7.

**Fig. 5:**
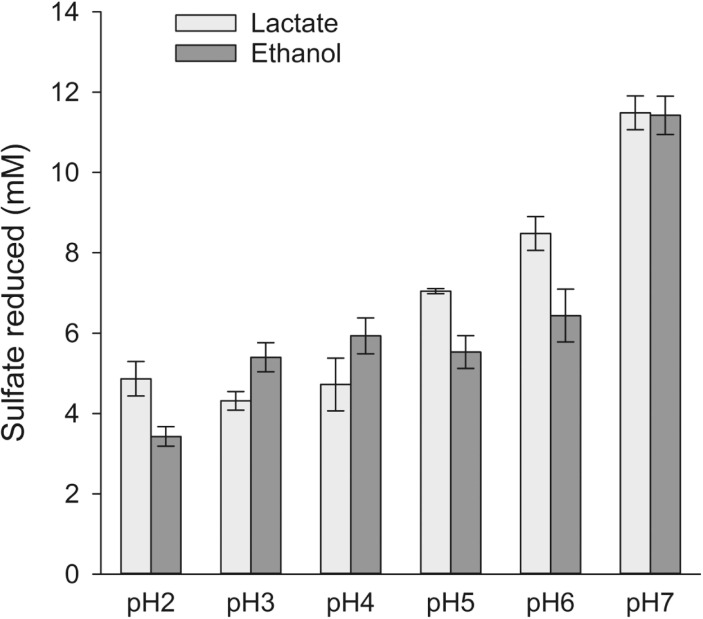
pH-dependent sulfate reduction by strain S4 in medium containing sulfate as the only terminal electron acceptor and lactate or ethanol as the electron donors.

Furthermore, the acid tolerance of strain S4 was examined in greater detail by using anoxic sulfate (28 mM) medium with pH ranging from 2 to 7. Two types of electron donors, lactate and ethanol (20 mM each) were used. The results (Fig. 5) indicated that strain S4 reduced sulfate at all the tested pH from 2 to 7. The strain showed best sulfate reduction at pH 7 with 11.5 mM sulfate reduced on the 15^th^ day of cultivation. At slightly acidic pH values of 5–6, the sulfate reduction was counted for 55–70% of that at pH 7, reaching 6.3–7.5 mM on the 15^th^ day of cultivation. At lower pH of 2–4, the sulfate reduction was still active, however at a rate equal to 30–50% of that at pH 7. Interestingly, even at such strongly acidic conditions of pH 2–4, strain S4 reduced sulfate with lactate (a dissociated electron donor) at a comparable level to that with ethanol (a undissociated electron donor).

### The Resistance to Heavy Metals

**Fig. 6:**
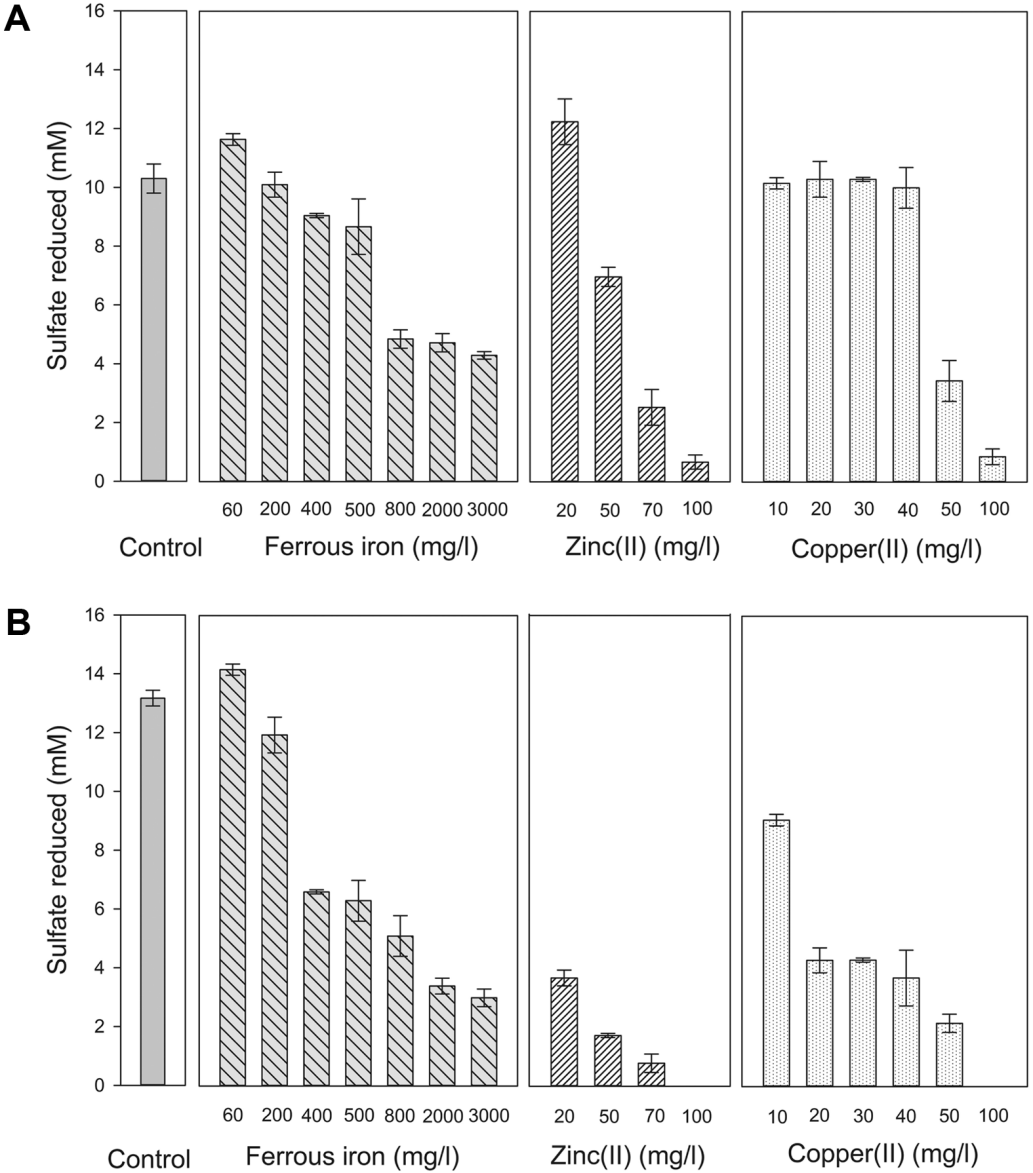
Inhibitory effect of different heavy metals on the sulfate reduction by strains S4 (A) and S10 (B). Controls were the cultures of strain S4 or strain S10 inoculated in medium without heavy metals.

The newly isolated strains S4 and S10 originated from the mud of an AMD storage pond; it was therefore expected that the strain would resist to high concentration of heavy metals. Indeed, the results (Fig. 6A) showed that strain S4 reduced sulfate actively in LS medium supplemented with various metals at very high concentrations. In the case of Fe^2+^, the sulfate reduction remained active in medium containing Fe^2+^ up to 500 mg/ l, just slightly decreased at the Fe^2+^ concentration above 500 mg/l and was inhibited by 50% at the Fe^2+^ concentrations of 800 mg/l and above, up to 3,000 mg/l. Zn^2+^ and Cu^2+^ showed higher inhibitory effects than Fe^2+^, as the sulfate reduction by strain S4 was decreased up to 90% at the concentration of 100 mg/l and above for both Zn^2+^ and Cu^2+^.

Comparing to strain S4, strain S10 was less resistant to heavy metals (Fig. 6B). This strain showed active sulfate reduction at Fe^2+^ concentration ≤ 200 mg/l, whereas at Fe^2+^ concentrations of 400 mg/l and above, the sulfate reduction decreased by 50–75%. Strain S10 was highly sensitive to both Zn^2+^ and Cu^2+^, as it stopped reducing sulfate at even a low concentration of 20 mg/l of Zn^2+^ or Cu^2+^.

### Growth Support for Other Common Neutrophilic SRB

**Fig. 7:**
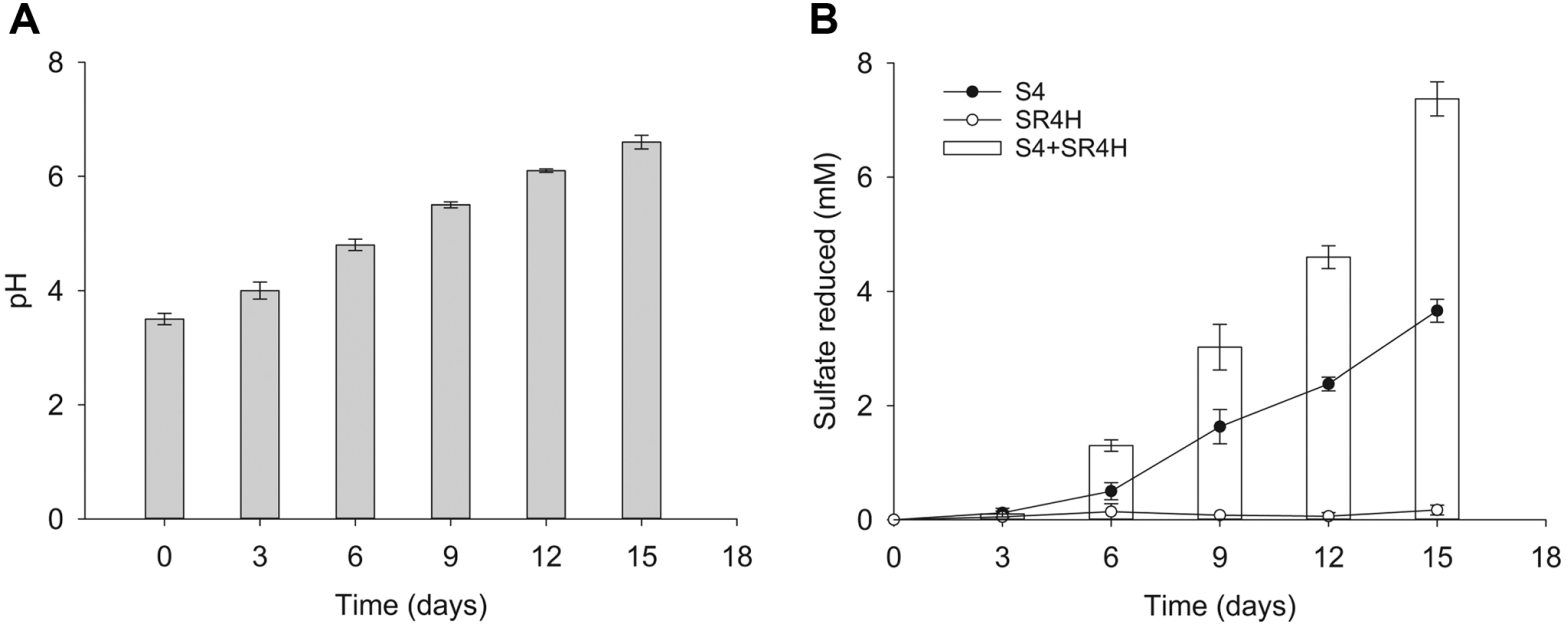
Effect of strain S4 in facilitating sulfate reduction of common neutrophilic, metal-sensitive strain SR4H in synthetic AMD. (**A**) – pH changing in synthetic AMD medium inoculated with strain S4 alone.; (**B**) – Sulfate reduction of (1) strain S4 alone, (2) strain SR4H alone and (3) co-culture of these two strains in AMD (after three days of co- inoculation). The longer strain S4 was pre-grown in AMD, the more suitable AMD environment became for strain SR4H, and the more the sulfate was reduced.

Results of the PCR-DGGE analysis showed that low pH condition in the enrichment culture led to accumulation of two main groups represented by the newly isolated acid- and heavy metal-resistant strain S4 and the neutrophilic, less metal-resistant strain S10. It is therefore speculated that the resistant species could play a pioneering role in the enrichment culture, as they started sulfate reduction and then rendered the extreme environment more suitable for the less resistant species to grow. Indeed, strain S4, while growing at low pH did increase the pH of the medium (Fig. 7A). To prove the leading role of this strain, a co-culturing experiment was carried out using the acid-tolerant strain S4 and a common neutrophilic strain *Desulfovibrio* sp. SR4H (VTCC 11270). Strain SR4H reduces sulfate visibly at pH 6 and above (Fig. S1). This strain alone in the synthetic AMD with pH 3.5 did not reduce sulfate (Fig. 7B). However, in co-culture with the acid-tolerant strain S4, active sulfate reduction was observed, at an even higher rate than sulfate reduction by strain S4 alone. It was also observed that the sulfate reduction in co-culture of these two strains was largely dependent on the pre-growth time of strain S4 in the synthetic AMD before strain SHR4 was added (Fig. 7B). In more detail, the highest sulfate reduction (7.8 mM after 3 days of co-inoculation) was observed when strain SR4H was added to 15-day-old culture of strain S4. The sulfate reduction was remarkably lower when strain SR4H was added to culture of strain S4 with shorter cultivation time, *i.e*. 12, 9, 6 and 3 days. If these two strains were inoculated together at the same time, no sulfate was reduced even after 3 days of incubation.

## Discussion

Treatment of acid mine drainage (AMD) by passive technology using a biological sulfate reactor has been proven for high efficiency and eco-friendliness [22]. Generally, it takes much time to establish an active SRB community that can adapt to the extreme condition in the AMD and perform sulfate reduction for the removal of heavy metals and increase the pH [23]. It has been shown that addition of acid-tolerant SRB to a sulfate-reducing reactor for AMD treatment more effectively stabilizes sulfate reduction [4]. However, with the native physiological trait of producing sulfide during the sulfate metabolism, acidophilic or acid-tolerant representatives are not common among SRB [5]. So far, only a few SRB strains were reported for acid-tolerant ability [7, 10, 24, 25]. In order to obtain acid-tolerant SRB for use in AMD treatment, we used mud of an AMD storage pond as inoculum for the enrichment process. The enrichment was carried out in anoxic lactate-sulfate medium at pH 5 and successfully yielded an SRB community dominated by two *Desulfovibrio* sp., represented by isolates S4 and S10. It was surprising that of the two isolates, only strain S4 was acid tolerant, whereas strain S10 appeared to be a common neutrophilic species. Strain S4 reduced sulfate at pH as low as 2, and thus became the second reported acid-tolerant SRB of the class *Deltaproteobacteria*. The first reported isolate was strain *Desulfovibrio* sp. TomC obtained from an acidic mining waste area in Siberia, Russia [10]. It was of interest that sulfate reduction by strain S4 at low pH was comparable if dissociated lactate or un-dissociated ethanol was served as the electron donor. This would make the strain better adapt to broad ranges of electron donors for sulfate reduction and become more competitive in AMD environment.

Physiological analysis also showed that strain S4 was resistant to various heavy metals, especially the highly toxic to living cell metals such as Cu^2+^ and Zn^2+^. Comparing to previous reports, the metal-tolerant ability of strain S4 was considerably high. Sani *et al*. (2001) demonstrated that strain *Desulfovibrio desulfuricans* G20 was inhibited by copper readily at concentration as low as 1.92 mg/l [26]. In another study, Utgikar *et al.* (2001) showed that a mixed culture of SRB was inhibited at 12 mg/l copper, 20 mg/l zinc [27]. Some other *Desulfovibrio* strains, i.e. *Desulfovibrio* sp. ATCC 49975 and *D. vulgaris* ATCC 29579 were inhibited at 9 mg/l copper, 20 mg/l zinc [28]. Evidentially, the tolerance of strain S4 to copper and zinc at the concentration of 100 mg/l was outstanding. This resistance level was even comparable to that observed in members of the spore-forming *Firmicute* group of SRB such as strain *Desulfosporosinus acididurans* M1T which was reported to resist copper at 64 mg/l [24].

The fact that acid-tolerant and acid in-tolerant SRB were accumulated in the enrichment culture at low pH was the first sign of the leading role of the acid-tolerant SRB in such a stressed environment like AMD. It is assumed that the acid-tolerant SRB such as strain S4 developed first in the enrichment culture, reduced sulfate to sulfide, thereby raising the pH in the medium. At the same time, the bacterium precipitated metals, created favorable conditions for other SRB, such as strain S10 which cannot tolerate low pH and high metal concentrations. This theory was experimentally proven by co-inoculation of the acid- and metal-tolerant strain S4 with the neutrophilic, metal-sensitive SRB strain SR4H from VTCC.

In the context of AMD treatment systems, the startup of treatment process would depend on such types of acid- and metal-tolerant SRB to a large extent. Their early colonization and sulfate reduction would help promote the remediation process. Once they start to reside and reduce sulfate in acidic condition, the extreme conditions in the environment, such as low pH and high concentration of heavy metal ions, would be decreased, establishing more suitable growth conditions for other SRB. In practice, the startup stage of AMD treatment process often takes time due to the lack of such acid-tolerant SRB. The acid-tolerant strain S4 obtained in this study would serve as an effective bacterial seeding to speed up the AMD treatment process in a sulfate-reducing bioreactor.

## Supplemental Materials



Supplementary data for this paper are available on-line only at http://jmb.or.kr.
