# Supplementary material for: Sulfate Reduction for Bioremediation of AMD Facilitated by an Indigenous Acid- and Metal-Tolerant Sulfate-Reducer
Source: J Microbiol Biotechnol. 2020 Mar 9;30(7):1005–12. doi: 10.4014/jmb.2001.01012 (PMC9728233; doi:10.4014/jmb.2001.01012)
Supplement: Supplementary file 1 [file JMB-30-7-1005-supple.pdf]

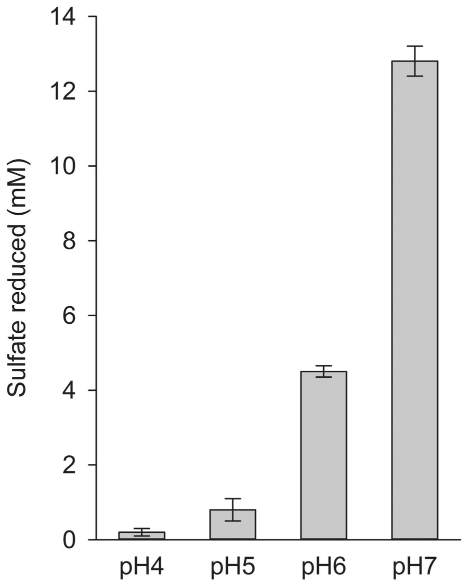


**Figure S1.** Sulfate reduction by strain SR4H at different pH values

Strain *Desulfovibrio* sp. SR4H (provided by Vietnam Type Culture Collection, VTCC 11270) is a common neutrophilic SRB. The strain visibly reduces sulfate at pH6 and above. No sulfate reduction was observed at pH4 and below.
